# Occurrence of 5-Hydroxymethylfurfural, Acrylamide, 3-Monochloro-1,2-Propanoldiol and Melamine in Infant Formulas: What Do We Know About These Compounds?

**DOI:** 10.3390/toxics13030161

**Published:** 2025-02-25

**Authors:** Xóchitl Yanine Méndez-Alvarado, María Magdalena Eréndira González-Tello, Jorge Luis Chávez-Servín, Karina de la Torre-Carbot, Teresa García-Gasca, Diana Beatriz Rangel-Peniche, Roberto Augusto Ferriz-Martínez

**Affiliations:** Faculty of Natural Sciences, Juriquilla Campus, Autonomous University of Querétaro, Av. de las Ciencias S/N, Juriquilla, Querétaro 76230, Mexicomaria.magdalenae.gonzalez@uaq.mx (M.M.E.G.-T.); karina.delatorre@uaq.edu.mx (K.d.l.T.-C.); tggasca@uaq.edu.mx (T.G.-G.); rangelp@uaq.mx (D.B.R.-P.); roberto.augusto.ferriz@uaq.mx (R.A.F.-M.)

**Keywords:** HMF, acrylamide, 3-MCPD, melamine, cyanuric acid, infant formulas

## Abstract

In the manufacture of infant formulas, from raw materials to the final product, the ingredients are subject to high temperatures which favor the formation of undesirable compounds, some of them from the Maillard reaction, such as 5-hydroxymethylfurfural (HMF) and acrylamide, and others from thermal processing, such as the compound 3-monochloro-1,2-propanoldiol (3-MCPD). Finally, there is also a risk that the product may be adulterated with undesirable components such as melamine and cyanuric acid. Due to the vulnerability of infants during the first stage of life, this review answers the main question: How much of these undesirable compounds are present in commercial infant formulas, and what do we know about them? Accordingly, the review is divided into three sections: (1) Maillard reaction products (HMF and acrylamide), (2) products contained in vegetable oils (3-MCPD), and (3) fraudulent and/or adulterant compounds (melamine and cyanuric acid). The objective is to report on the occurrence of HMF, acrylamide, 3-MCPD, melamine, and cyanuric acid in infant formulas in order to support more solid public health policies related to infant feeding. These undesirable compounds represent a risk to infants, possibly contributing to kidney and neurological damage and causing mutations that increase the development of childhood cancer. Therefore, it is necessary to promote breastfeeding and establish stricter controls, with scientific evidence on the effects of HMF, acrylamide, 3-MCPD, melamine, and cyanuric acid in infant formulas to reduce their short- and long-term effects on infants’ health.

## 1. Introduction

Because of its many health benefits, breastfeeding is universally recognized as the best solution to the problem of infant nutrition. Indeed, the World Health Organization (WHO) recommends that breastfeeding be offered exclusively for the first six months of life and then continued as a supplement until the second year [1]. Breastfeeding has been suggested as the optimal diet for premature and extremely premature babies [2,3].

Breast milk is a dynamic and complex fluid that supplies the infant with all its initial nutritional requirements and is also essential for optimal growth and development [4]. More than a thousand components have been identified in breast milk, including trace elements, growth factors, and various bioactive compounds [5,6,7]. Breastfeeding is recognized for its importance in the immunological and neurological development of babies due to the presence of these multiple compounds [8,9]. When breastfeeding is not possible, the food industry has been developing formulas designed to replicate breast milk. Infant formulas are defined as food used by infants during the first months of life and satisfying themselves the nutritional requirements of such infants until the introduction of appropriate complementary feeding [10]. Most commercial infant formula brands come in powder form and are made primarily from cow’s milk. They are typically manufactured using specific combinations of proteins, fats, carbohydrates, vitamins, minerals, and other minor components. In standard infant formula production, the raw material mixture is blended, pasteurized, homogenized, condensed, and spray-dried or sterilized. During these treatments and storage, redistribution and interactions between the major components can give rise to undesirable compounds [11]. In these processes, interactions between compounds, such as protein-carbohydrate, protein-protein, and protein-lipid, are favored, which can lead to the formation of undesirable and potentially toxic compounds [12]. For this reason, the World Health Organization [13] published an International Code of Marketing of Breast-milk Substitutes that would regulate the marketing and mass consumption of infant formulas, recommending that their use be restricted to necessary cases and where breast milk is not the main source of nutrition [13]. The WHO, UNICEF, and the International Network of Baby Food Action Groups (IBFAN) have reported that many mothers continue to receive information promoting the use of infant formulas without any health consequences. Of 194 countries evaluated by the WHO, only 79 have banned the marketing of infant formulas in health centers [14]. The use of infant formula is more prominent in more economically stable countries, causing a threat to the practice of breastfeeding and promoting infant mortality [15]. Furthermore, in developing countries, breastfeeding may give way to the use of infant formulas due to the lack of information and the need for mothers to work outside the home [16]. A number of diseases have been related to the consumption of infant formulas, most prominently diarrhea and pneumonia, which cost the health system 71.45 million dollars annually (mda) in high-income countries, between 430.03 and 604.46 mda in middle-income countries, and 40.88 mda in low-income countries, in all cases a significant economic impact. In addition, feeding a child with infant formula can consume an average of 6.1% of family income, more in the case of families with a lower socioeconomic level. Infant deaths due to a lack of prevalence in breastfeeding in children under 6 months are significant. An estimated 16,146 deaths in China, 99,552 in India, 15,028 in Indonesia, 2360 in Mexico, and 103,742 in Nigeria are attributed to the lack of breastfeeding. These data reflect a negative impact on infant health from the consumption of infant formula [17]. Other associated risks include an increase in gastrointestinal diseases, infectious diseases, poor development of the central nervous system, and adverse effects related to contamination of infant formula [18]. Given the susceptibility of infants to epigenetic mechanisms during the first stage of life (e.g., alterations in DNA gene expression because of environmental exposure [19,20,21], it is critical to assess available scientific information on the possible occurrence and impact of undesirable compounds in infant formulas.

These have been the subject of considerable research using a variety of analytic methodologies, with a focus on the analysis of undesirable compounds [22,23,24]. Based on their results, various methodologies have been developed to detect undesirable compounds in milk formulas [25], while regulatory bodies have been establishing minimum quality parameters for baby food formulas [26,27]. The World Health Organization (WHO) and the United Nations Food and Agriculture Organization (FAO) have developed a set of international standards called Codex Alimentarius, which aims to protect children’s health and ensure fair practices in the food trade. The “Standard for Infant Formula and Formulas for Special Medical Purposes Intended for Infants, CXS 72-1981” establishes that breast milk substitutes must scientifically demonstrate their safety and comply with the limits for contaminants established in the “General Standard for Contaminants and Toxins in Food and Feed (CXS 193-1995)” [28]. For its part, Regulation (EU) No 609/2013 of the European Parliament and of the Council establishes that infant formulae must be guaranteed to be safe and suitable and to meet the nutritional needs of healthy infants. Furthermore, all ingredients used in their manufacture must be suitable for infants and it is the responsibility of the company to present evidence to this effect, in addition to complying with the nutritional values presented in the regulation [29](Commission Delegated Regulation (EU 2016/127). The FDA requires companies to notify it when marketing a new infant formula. The nutrients contained in the infant formula must be specified, and the product must be subject to sanitary controls to guarantee that it is not contaminated and to ensure its safety. In addition, controls must be implemented to identify the presence of microorganisms [30]. These international bodies do not set maximum levels for compounds generated in the production and storage of infant formulas. It is therefore up to each country to develop rigorous national standards that guarantee the protection of infant health through strict analytical control.

Infant formulas may become unstable because of factors that induce changes in their formulation, such as the Maillard reaction, derived from the content of lactose or reducing sugars, the amount of lysine, and the high temperatures used in manufacturing and storage [31,32]. The quality of the raw materials used is also relevant since these may contain substances that are harmful to the infant, such as melamine (MEL) and cyanuric acid (CYA) [33]. Thus, makers of infant formula face a dual technological challenge: achieving stability both in its composition and during its storage.

Most studies of the Maillard reaction focus on evaluating production processes and storage conditions (storage time/temperature conditions/raw materials) but say little about the possible adverse effects of sustained consumption by infants [11].

This paper reviews studies that have reported the content of undesirable compounds derived from the Maillard reaction during the manufacture of infant formula and the presence of other compounds that are present or developed during their production or storage. Our analysis is divided into three sections: (1) Maillard Reaction Products (HMF and acrylamide), (2) Undesirable vegetable oil products (3-MCPD (3-Monochloro-1,2-propanoldiol), and (3) Fraudulent content or adulteration (melamine and cyanuric acid). We also review information about the health effects of these compounds.

## 2. Methods

Our search reviews the literature on the content of undesirable compounds in infant formulas, specifically HMF, Acrylamide, 3-MCPD, Melamine, and cyanuric acid. Platform sources consulted include: PubMed, ScienceDirect, Google Academic (in English or Spanish, and regardless of country of origin) from 2000 to 2024. We followed the Preferred Reporting Items for Systematic Reviews and Meta-Analyses (PRISMA) guidelines. Duplicate articles and those in which the undesirable compound was deliberately added to infant formula for the purpose of evaluating the analytical methodology or studying possible chemical interactions were excluded. The search keywords were: HMF in infant formulas, Acrylamide in infant formulas, 3-MCPD in infant formulas, Melamine, and cyanuric acid in infant formulas. These words had to be contained in the title, abstract, and/or results for inclusion. All published adverse effects were linked to the undesirable compounds analyzed.

## 3. Results

**Figure 1:**
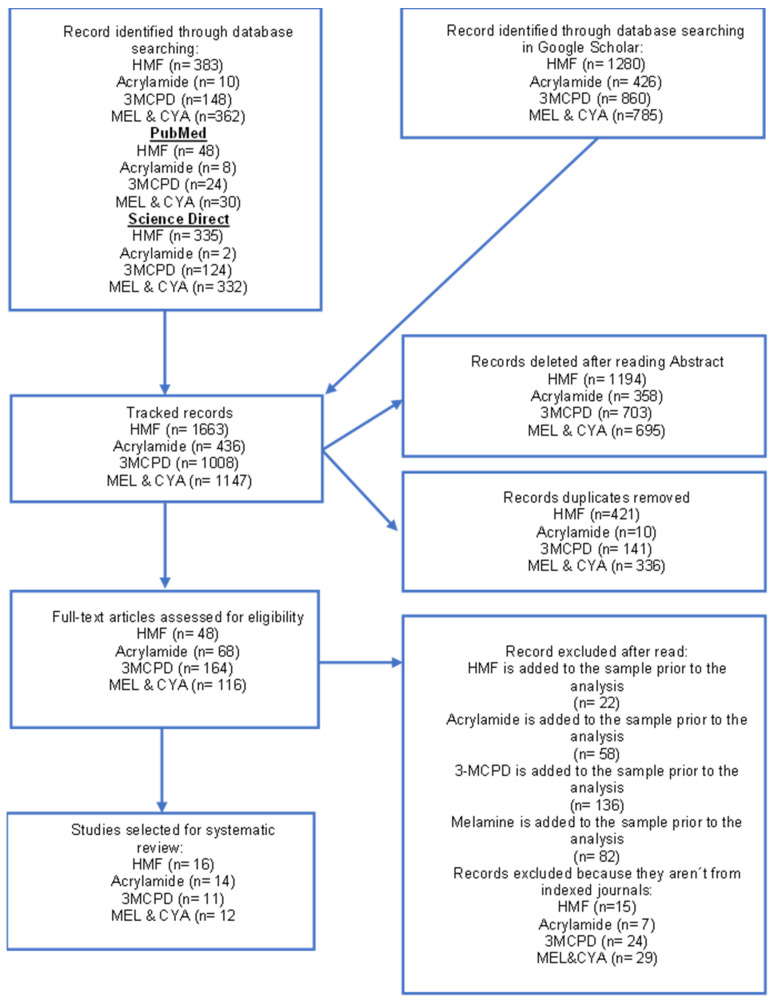
Scheme of Preferred Reporting Items for Systematic Reviews (PRISMA). HMF, Hydroxymethyl furfural; 3-MCPD, 3-Monochloro-1,2-propanediol; MEL, Melamine; CYA cyanuric acid.

A total of 4254 articles were initially identified: 1663 for HMF, 436 for Acrylamide, 1008 for 3-MCPD, and 1147 for Melamine and cyanuric acid (MEL and CYA). After applying our review criteria, eleven articles on HMF, four on acrylamide, five on 3-MCPD content, and four on Melamine and cyanuric acid in infant formula were included (Figure 1).

### 3.1. Products of the Maillard Reaction

Infant formulas are prone to developing furfural compounds, which are undesirable compounds derived from the Maillard reaction, a non-enzymatic browning reaction that produces melanoidins. It occurs between carbonyl groups of carbohydrates (reducing sugars) and groups of amino acids, peptides, or proteins. It is produced by several factors such as a change in pH, exposure to high temperatures, water activity, the type of reactive elements, and the presence of metal ions [34,35,36]. These compounds are formed during heat treatment used in formula production and/or during storage [37]. Dairy formulas contain lactose (a reducing sugar) and lysine (an amino group), which are the main compounds involved in the initial stages of the Maillard reaction. These produce lactulosyl-lysine, which in intermediate stages of the Maillard Reaction result in furfural compounds. Furfurals can be produced in two ways: via the Amadori compounds (mainly ∈-N-deoxylactulosyl-L-lysine) as part of the Maillard reaction, by enolization under acidic conditions, or via the isomerization of lactose known as the Lobry De Bruyn-Alberda van Ekenstein transformation and subsequent degradation reactions [38,39,40].

The main compound reported in infant formulas is 5-hydroxymethylfurfural (HMF) [32]. This compound serves as an indicator of food damage and as an assay of the Maillard reaction [41]. The formation of acrylamide via the Maillard reaction from free asparagine and carbonyl sources was reported as early as 2002 by the Swedish National Food Administration [42].

#### 3.1.1. Hydroxymethyl Furfural (HMF); CID: 237,332

**Figure 2:**
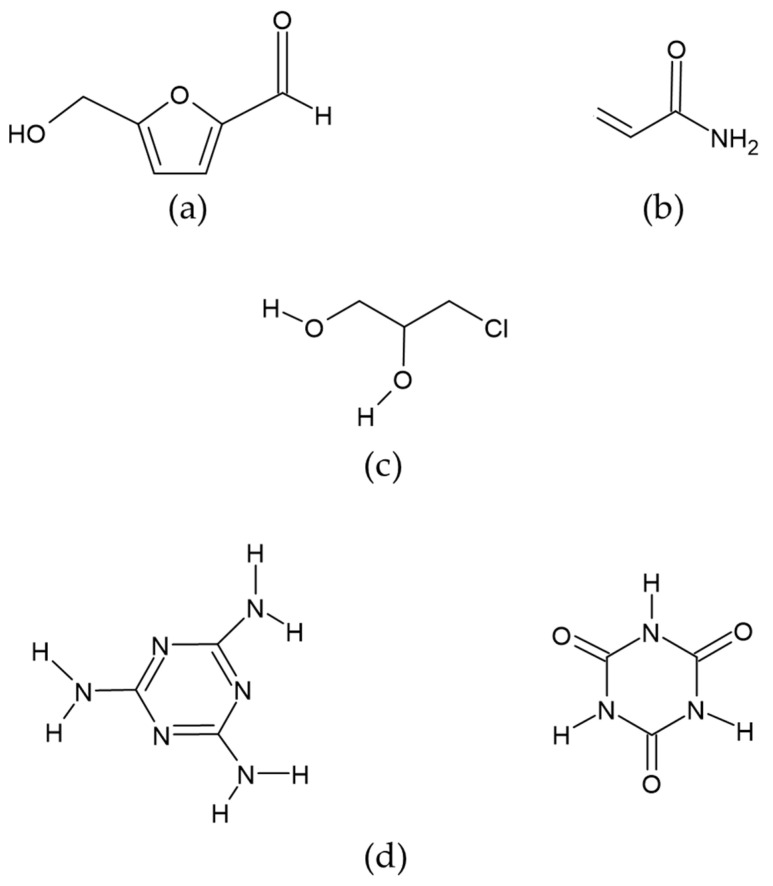
Chemical structure of compounds: (**a**) Hydroxymethyl furfural (HMF), (**b**) Acrylamide, (**c**) 3-MCPD (3-Monochloro-1,2-propanediol), (**d**) Melamine (MEL) and cyanuric acid (CYA).

This is a six-carbon heterocyclic organic compound containing both aldehyde and alcohol (hydroxymethyl) as functional groups. It has a central ring of furan residues linked to formyl groups in the second position and a hydroxyl in the fifth (Figure 2a). It is considered the most important intermediate product in the Maillard reaction. It is prevalent in food products that are exposed to thermal treatments (baking, cooking, pasteurization, etc.), making them more edible and preserving them, either by reducing the microbial load and/or by eliminating the enzymatic activity [43,44].

It has been estimated that daily human consumption of HMF ranges from 30–150 mg/person/day when very high doses of exposure in food, although the Federal Institute for Risk Estimation proposes a food consumption range of 4–30 mg of HMF/person/day [45,46]. There is preliminary evidence that when consumed orally by a person, 99.25 % of HMF is not excreted in the urine and remains in the body [47]. A rapid absorption at the gastrointestinal level and association of its metabolites with incipient aberrant cell foci at the colon level, and a possible association with colon cancer, have also been documented. One of the main metabolites studied in animal models of rats and mice is 5-sulfoxymethylfurfural (SMF), characterized by the in vivo transformation of HMF to SMF through sulfonation of the active allylic hydroxyl group. This is initiated by sulfotransferases and a sulfo-group donor, 3-phosphoadenosine-5-phosphosulfate (PAPS). This unstable metabolite, SMF, is detectable in blood, and its biotransformation after exposure to HMF has been studied in vivo [48,49].

**Figure 3:**
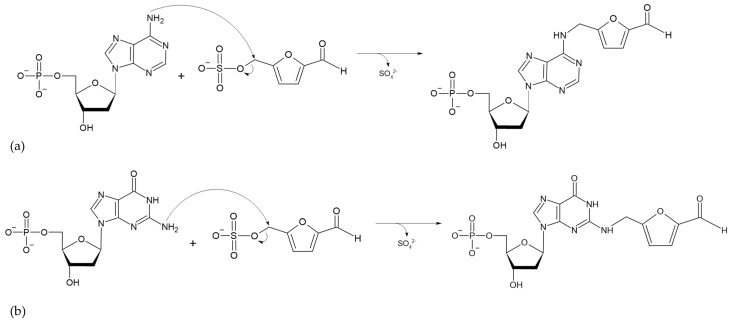
Reaction mechanism for the formation of DNA adducts (**a**) N6-FFM-dAdo and (**b**) N2-FFM-dGuo, from SMF by a nucleophilic reaction (Modified from [53]).

**Figure 4:**
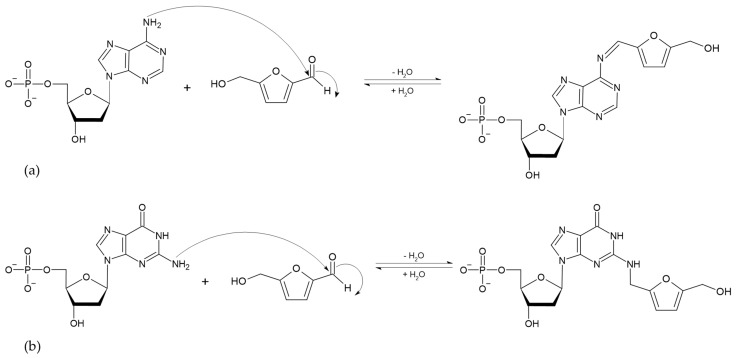
Reaction mechanism of hexocyclic amino group condensation for the formation of (**a**) N6-HMF-dAdo and (**b**) N2-HMF-dGuo DNA adducts from HMF (Modified from [53]).

The absorption of HMF through the cell membrane of enterocytes, without saturating the system, is up to 65 mg/L. Although the mechanism by which HMF enters the cells is not clear, it is believed to be by passive diffusion or by protein-mediated transport [50]. Once HMF enters the enterocytes, the sulfotransferase SULTA1A1 and the cofactor PAPS transfer a sulfo group, giving rise to the formation of SMF, through a phase II reaction [51,52]. Once SMF enters the nucleus, it is able to interact with DNA to form adducts. Among the main DNA adducts formed are those resulting from the nucleophilic reaction at the benzyl carbon of SMF, which result in two adducts, N^2^-((2-formylfuran-5-yl)methyl)-2′-deoxyguanosine (N^2^-FFM-dGuo) and N^6^-((2-formylfuran-5-yl)methyl)-2′-deoxyadenosine (N^6^-FFM-dAdo) (Figure 3); and those resulting from the condensation of a hexocyclic amino group of a deoxyadenosine, which results in adduct N^6^-((2-hydroxymethylfuran-5-yl)methyl)-2′-dAdo (N6-HMF-dAdo) and a deoxyguanosine resulting in N^2^-((2-hydroxymethylfuran-5-yl)-methyl)-2′-dGuo (N2-HMF-dGuo) (Figure 4) [53].

Several studies have reported the presence of potential HMF in infant formulas (Table 1). When infant formulas were stored and tested at different temperatures over time [54], an increase in HMF was found as the storage temperature increased. A distinction was made between free and potential HMF. To determine potential furfurals, samples were hydrolyzed to release HMF (the formation of this furfural from Amadori’s compound is induced under acidic conditions). Thus, the potential HMF level represents the sum of HMF precursors (protein-bound HMF, such as Amadori products, de novo, or reducing sugar HMF) and free HMF. Free HMF is determined without performing the acid hydrolysis step [32].

In 20 commercial brands of infant formulas tested after opening the packets and storing 30 and 70 days at room temperatures 23–25.5 °C within the first 3–5 months of their shelf life, mean initial potential furfural values (HMF + 2-furaldehyde) were 1115.2 µg/ 100 g [11]. These values were 1157.6 µg/ 100 g and 1344.5 µg/100 g, at 30 and 70 days later, respectively. Values for potential HMF were higher in the studied formula brands, ranging from 417–2141 µg/100 g immediately after opening the packets. After 70 days of storage, the values of potential HMF ranged between 462 and 3121 µg/100 g. The difference between the values of potential HMF in the brands of infant formulas proved to result mainly from the composition, the casein/serum protein ratio, the thermal treatments used during manufacturing, and the storage conditions. A similar increase in the content of potential furfurals secondary to the Maillard reaction was seen in powdered milk-based infant formulas after package opening.

In Spain, Sabater et al. compared the Maillard reaction in commercial infant formulas with and without supplementation with prebiotics. Furosine content ranged from 94–1226 mg/100 g protein (without prebiotics) and 315–965 mg/100 g protein (prebiotics). Likewise, they reported increased Maillard-reaction-related HMF in all the infant formulas analyzed in a range of 62–510 µg/100 g of product as a function of storage time. There were no statistically significant differences between formulas supplemented and not supplemented with prebiotics [58]. In recently manufactured milk-based formulas, HMF values are higher in formulas that use higher temperatures in their production processes and those stored at high temperatures for long periods. These conditions promote the Maillard reaction in infant formulas and may account for different concentrations of HMF [56,59,62]. In 2020, Czerwonka et al. [61] evaluated the effects of 5-HMF concentration secondary to fat content, lactose or its hydrolysis products, and type of preservation in cow’s milk and modified infant milk. The study revealed an average 5-HMF content of 230 µg/100 g in powdered infant formula.

At the nutritional level, the consequences of the Maillard reaction in infant formulas include the loss of bioavailability of lysine, an amino acid essential for infant growth and development. HMF is hepatotoxic and associated with the deterioration of liver functions and the development of hepatocellular adenomas [63,64]. Bauer-Marinovic, et al., 2012 studied these effects extensively, and both confirm and provide a possible mechanism for the heptocarcinogenicity of HMF [65]. They studied the effects of intraperitoneally administered HMF at different doses in wild-type and transgenic mice. In the first experiment, 35 mice were administered a dose of 250 mg/kg, and most of them died within 5 to 11 days due to massive damage to the proximal tubules of the kidneys. In the following experiment, intraperitoneal SMF was administered on two occasions at 7-day intervals, and the mice were sacrificed eight weeks after treatment to assess the effects on liver and kidney tissues. In this last experiment, the mice were divided into four subgroups: doses of 62.5 mg/kg and 125 mg/kg of SMF, doses of 10 mg of azoxymethane/kg, and 35 mice treated with a saline solution that served as a control group. It was determined that the lethal dose was above 125 mg/kg up to 250 mg/kg and that progressive but constant administration also caused damage to specific tissues in the liver and kidneys. Analysis revealed serositis, hepatic and renal fibrosis, hepatomegaly, hepatic necrosis, nodular hyperplasia, acute necrosis in adipose tissue, and formation of adhesions of the liver to the diaphragm or to the right kidney. The authors suggest an affinity of SMF for specific receptors in proximal tubules that prevent it from being eliminated, reabsorbed, or remetabolized, causing liver damage. Monien et al. (2012) studied the reactivity of DNA to HMF by incubating a plain DNA sample with HMF at a concentration of 4 mM with NaBH_3_CN added to reduce imines [53]. This experiment verified the hypothesis that human SULT1A1 bioactivates HMF through sulfoconjugation to a sulfate ester that binds to DNA and is mutagenic. This mechanism represents a plausible reason for hepatocarcinogenicity [53,66]. Wang and colleagues (2019) studied the cytotoxicity of HMF on three cell lines of human gastric epithelial cells and human vascular endothelial cells. The authors reported injury to gastrointestinal cells at low concentrations of 32 mM. Likewise, more than 95 % of the cells died after exposure of 28 and 24 mM of HMF for 24 and 48 h. The same effect occurred in vascular endothelial cells exposed to 24 and 12 mM. The authors note that foodborne HMF is a potential hazard and can easily form adducts with amino acids in foods during temperature processing or during the digestion process. They conclude that HMF is prone to reactivity to pH changes and that the level of gastrointestinal absorption is not fully known and merits further study [67]. Pastoriza de la Cueva et al. [68] determined the presence of N^2^-FFM-dGuo and N^6^-FFM-dAdo adducts in a sample of leukocyte DNA from children with a mean age of 10.7 ± 0.5 years. Their results report 4.67 ± 0.20 (amount per 10^8^ DNA nucleosides) of N^2^-FFM-dGuo and 0.50 ± 0.20 (amount per 10^8^ DNA nucleosides) of N^6^-FFM-dAdo. They conclude that the presence of DNA adducts in children is statistically correlated (*p* < 0.05) with an intake of foods containing HMF, indicating that constant exposure to HMF and its derivatives from an early age would have a significant impact on health. DNA adducts can induce mutations, triggering the development of adenomas in the small intestine, at an HMF consumption of 500 mg/kg of weight in mice [69], in addition to generating other negative changes in processes related to DNA transcription and replication, inducing one or more mutations, mainly for the activation of oncogenes such as H-ras and K-ras and changes in the tumor suppressor gene p53 [70]. HMF neurotoxicity has also been studied in animal models, where suppression of exploratory behavior, inability to move normally, grand mal seizure states, and unconsciousness have been reported. The same study reported an early onset of puberty and a significant reduction in ovarian reserve in female animal models [48].

With daily consumption of HMF present in food in children, this compound can be found in plasma from 30 min, reaching its peak at 90 min. In the liver, it is detected at a maximum peak of 60 min, and in the kidneys at 120 min [68]. Prior et al. [71] evaluated the elimination time of HMF metabolites in women’s urine after the consumption of 3944 µmol and 486 µmol of HMF present in plum juice and dried plums, respectively. They reported that in the first 6 h, the elimination of HMF metabolites in urine was 46.2 % for a concentration of 3944 µmol, and 14.2 % for a concentration of 486 µmol. Therefore, the authors suggest that HMF is rapidly metabolized before being excreted in urine. Hardt-Stremayr et al. [72] identified the compounds of HMF metabolism in human urine using High-Performance Liquid Chromatography (HPLC). The administered dose was 240 mg of HMF in 30 mL of solution. Their results show that the presence of HMF was not detected in the urine samples. However, the highest concentrations of its metabolites were identified at 2 h after HMF administration and progressively decreased until 48 h, with 90 % elimination.

The range of HMF intake in 6-month-old infants fed exclusively milk-based formulas has been estimated to be between 0.63 and 3.25 mg of HMF per day. This value was obtained from studying HMF content in various commercial infant formulas from several countries, considering the average minimum and maximum values of the brands of formulas studied [11] Also, the Scientific Panel on Food Additives, Flavors, Processing Aids, and Food Contact Materials (AFC) estimated a dietary HMF intake of approximately 1.6 mg/person per day based on a modified Theoretical Added Maximum Daily Intake (mTAMDI) approach [73]. These values are far from the threshold of concern of 540 micrograms/person/day derived from an extensive database on chronic and subchronic diseases in animal studies [74]. However, the main concern regarding HMF consumption centers around its conversion to sulfoxymethylfurfural (SMF) by sulfotransferases, a mutagenic compound and therefore a potential risk for infants who consume infant formulas containing HMF [11,73]. The NTP’s NOAEL for HMF is a conservative estimate. This means that the actual NOAEL for HMF is probably higher than 500 mg/kg/day for oral exposure and 200 mg/kg/day for inhalation exposure [63]

#### 3.1.2. Acrylamide; CID: 6579

Acrylamide is a solid monomer whose chemical structure comprises a polar amide group and a vinyl function that allows it to polymerize. It is also known as ethylcarboxamide, vinylamide, or 2-propanamide, which occurs as white crystals. It is a reactive alpha, beta unsaturated carbonyl molecule; with a molecular weight of 71.08 MW and a molecular formula of C_3_H_5_NO (CH2=CHCNH2) [75] (Figure 2b). It is a byproduct of the Maillard reaction between reducing sugars (glucose and fructose) and free asparagine. It frequently occurs in starchy foods subjected to heat treatments above 120 °C. Brathen et al. [76] evaluated the formation of acrylamide in relation to time and temperature in conventional bakery ovens used to manufacture cereal-based food products. Their results indicate that, in breads with a humidity of less than 4 %, the concentration of acrylamide increased. However, with increasing baking times, the concentration decreased. The authors conclude that, in dry systems, acrylamide concentration peaks at a temperature range of 190–210 °C. Ahrné et al. [77] reported that acrylamide begins to form in the temperature range of 120–130 °C, with a humidity between 4–2%, reaching a concentration of 230 ± 18 µg/Kg in the bread crust. However, when the baking temperature is increased to 231 °C, the acrylamide concentration decreases to a concentration of 135 ± 17 µg/kg. Despite this decrease, prolonged baking time causes the bread to become inedible, with a dark color and undesirable sensory properties. The thermal process used in the production of infant formulas favors the increase in acrylamide levels. Factors such as temperature, time, and humidity have a significant impact on the mechanisms for the formation of acrylamide.

The presence of acrylamide is recognized in foods such as potatoes and cereals. However, its content in infant formulas has recently come to light, and analysis has shown that intake during the first year of life may be significant (Table 2). This is because the main foods consumed by infants at this stage are powdered formulas, cereals, and porridge—three products that contain considerable amounts of acrylamide [78]. Acrylamide is not a natural component of breast milk; when present, it is produced by the mother’s consumption of fried foods or foods containing significant amounts of acrylamide, which are transferred to breast milk. In infant formula, however, amounts of 0.6 and 0.7 µg/kg were reported in 5 out of 8 products, with the reference value being 2.5 µg/kg. In Sweden, Fohgelberg and colleagues compared breast milk and infant formulas whose main ingredients were dehydrated wheat, skim milk, and vegetable oils, reporting much lower levels of acrylamide in breast milk than in the formulas. Although these findings appear to indicate a minimal risk of exposure to acrylamide, infants are also exposed to other foods containing acrylamide, such as purees and cereal pastes [79] In Poland, baby food products and dietary exposure to acrylamide were analyzed in infants aged 6–12 months. The authors suggest that the exposure concentration of acrylamide indicates significant exposure, considering that this is an early stage of life, and the intake of acrylamide throughout life is at a level of 0.08 µg/kg/day [80]. In a study by Sirot et al. [81], dietary exposure to acrylamide for three consecutive days was analyzed in 705 non-breastfed children under three years of age. In this study, dietary exposure to acrylamide was of sufficient concern to necessitate management measures to reduce these exposure levels. In 2021, Ghiasi and colleagues [82] evaluated the acrylamide content of powdered infant formulas that included three brands for three age groups. The results showed that in all three age groups, infants faced a high risk of toxicity from exposure to acrylamide and that its occurrence varied depending on other factors such as temperature and heating time used in the production process.

**Figure 5:**
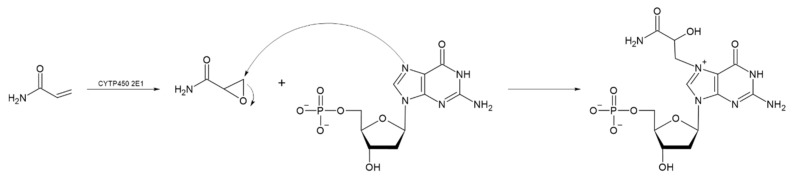
Reaction mechanism for the formation of glycidamide, a compound formed by epoxidation of acrylamide, and subsequent formation of the DNA adduct called N^7^-GA-dG. (Modified from [91]).

Acrylamide is metabolized to reactive glycidamide during epoxidation. This acrylamide metabolite becomes genotoxic by binding to DNA [86,87]. Glycidamide can be formed at a higher rate in children than in adults, and its detoxification is less effective in children, increasing its potential toxicity. Children generally have intakes 2 to 3 times that of adults relative to body weight, with significant intake in toddlers and infants [84,88]. According to the US Environmental Protection Agency (EPA), the no-observed-adverse-effect level (NOAEL) for acrylamide in rats is 0.2 mg/kg/day for oral exposure [73]. Various methodologies have been used to study its health effects [89] concluding that as acrylate, it has possible carcinogenic effects but with no clear threshold, which means that exposure to a single molecule can trigger a potentially carcinogenic biological process. Besaratinia and Pfeifer [90] studied the in vitro effect of glycidamide on acrylamide-induced mutagenesis in embryonic fibroblasts in transgenic mice and in TP53 cells. Their results indicate that glycidamide has a greater mutagenic capacity than acrylamide, although both compounds have the capacity to form DNA adducts (Figure 5). A study by Watzek et al. [91] evaluated the dose-response relationship with acrylamide and the formation of DNA adducts in Sprague-Dawley rats. Their results showed that from a concentration of 10 µg/kg of body weight, there was a significant increase in the levels of N^7^-GA-Gua N7 in the liver, kidneys, and lungs.

**Figure 6:**
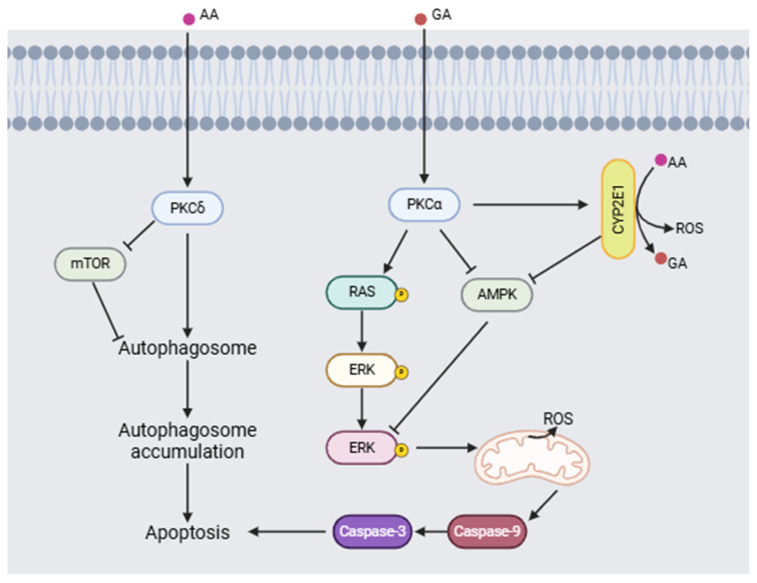
Proposed neurotoxic mechanism for acrylamide (AA) and glycidamide (GA) inducing apoptosis (modified from [92,93]). (Image created using BioRender.com (BioRender V2025)).

Triningsih et al. [92] evaluated the molecular mechanisms of acrylamide and glycidamide neurotoxicity in an in vitro PC12 cell model. Their results show that when acrylamide is metabolized to form glycidamide, it causes an increase in the concentration of reactive oxygen species (ROS), a process mediated by cytochrome 2E1 (CYP2E1), which makes ROS a key factor in neuronal damage. In addition, the presence of antioxidants did not reduce the damage induced by acrylamide. Furthermore, acrylamide and glycidamide promote the activation of protein kinase C (PKC) and subsequent phosphorylation of ERK, as well as a decrease in the activation of AMPK and mTOR, causing activation of the signaling pathway involved in cell death (Figure 6).

Acrylamide has also been associated with neurotoxicity in the sensitive, motor, and autonomic nervous systems. The pathogenesis that develops from an accumulation of acrylamide is characterized by axonal degeneration and constant inflammatory processes in the axons. Morphologically, worn neurofilaments and damage to specific receptors such as Pacinian corpuscles, annulospiral terminals, and Golgi tendons have been observed, producing inhibition of neurotransmission. These effects have been studied in mammalian animal models, primarily rodents, at exposure rates of 10–50 mg/kg/day. The exposure rates also showed neurological changes at different times. For example, during 11 days at 50 mg/kg, the neurotoxic effect is similar to exposure for 40 days at 21 mg/kg, reaffirming that acrylamide damage is caused by accumulation. The clinical signs for neurotoxicity are described as degeneration of the dorsal spinocerebellar tract, generating ataxia and loss of balance; at the motor level, as musculoskeletal weakness. In the autonomic system, urinary retention, baroreceptor dysfunction, and impaired vasomotor control are reported [94,95]. Studies in humans that describe neurotoxicity—there have been specific populations studied for exposure to acrylamide—report muscle weakness and neuropathies of the autonomic system like those reported in animal models. Thus, toxicological studies carried out in animal models have observed the carcinogenic, genotoxic, neurotoxic, immunological, and reproductive health effects of acrylamide, which is classified by the International Agency for Research on Cancer (IARC) as a probable “Group 2 A” carcinogen [96] Despite such concerns, the agencies responsible for determining toxicological limits for each type of food, have not yet defined the maximum acceptable limits of acrylamide in different types of food. It is urgent that limits and reference values be established for the food industry to apply in order to prevent harmful effects on infant health.

The study of toxicokinetics and metabolism of acrylamide and glycidamide have been widely studied due to their presence in foods and their adverse effects on human health. Vikström et al. (2011) studied the concentration-time relationship of acrylamide in humans who consumed a diet high in this compound [97]. The study design consisted of an initial four-day phase for the consumption of a dose of 11 µg/Kg of body weight (high intake), and subsequently a 28-day period with a dose of 2.5 µg/Kg of weight (medium intake). They reported that the reactivity of glycidamide was greater compared to the reactivity of acrylamide, meaning that more easily forms hemoglobin adducts. The determined area under the concentration-time curve (AUC) relationship in the blood reflects the differences in absorption and detoxification, indicating that the half-life of acrylamide is longer than observed in animal models. On the other hand, the AUC-acrylamide and AUC-glycidamide ratios were 200 and 50 nanomolar hours (nMh)/µg/kg of weight, respectively, indicating that in food intake, the exposure to acrylamide will be higher than to glycidamide. Fennell et al. (2006) evaluated the metabolism of acrylamide to glycidamide in people exposed to acrylamide [98]. Subjects were divided into three groups, with oral doses of 0.5, 1.0, and 3.0 g/kg of acrylamide-^13^C_3_, and urine samples were subsequently collected at 0–2, 2–4, 4–8, 8–16, and 16–24 h. The authors reported that the recovery rate of metabolites was 39.9 % of the administered dose and estimated the rate constant for acrylamide elimination to be between 0.21 and 0.26 h, or 3.1–3.5 h of its half-life. However, they were unable to quantify urinary glycidamide by liquid chromatography-tandem mass spectrometric (LC-MS/MS). Luo et al. (2022) studied the toxicokinetics of acrylamide and glycidamide in the blood of a rat model [99]. Doses of 0.1 mg of acrylamide/kg of weight (low dose) and 5 mg/kg (high dose) were administered. Their findings show that glycidamide had a half-life of 4.93 h, with a plateau phase of 3–6 h, suggesting a metabolic saturation of CYP2E1. In addition, acrylamide detoxification occurred through the formation of the acrylamide-GSH conjugate at 8 h. These studies highlight the importance of understanding the dynamics of acrylamide metabolism, from how it is captured to its elimination, in order to estimate its impact on human health.

### 3.2. Products from Vegetable Oils

#### 3-MCPD (3-Monochloro-1,2-Propanediol); CID: 7290

3-MCPD (3-Monochloro-1,2-propanediol) is a glycerol chlorohydrin formed when a chlorine atom replaces a hydroxyl group in a glycerol molecule. It is a non-volatile compound with a high boiling point (213 °C), soluble in water, and highly soluble in fats (Figure 2c). There are three routes of formation for this compound. The first is acid hydrolysis-a reaction of hydrochloric acid with residual vegetable oil; the second is during industrial thermal processing or domestic cooking—it is formed from lipids and sodium chloride—; and the third is the release of free 3-MCPD from its esterified (bound) form, which occurs by lipase-catalyzed hydrolysis in the human gastrointestinal tract during digestion [100]. The European Union’s Scientific Committee on Food established a maximum tolerable daily intake of 2 µg/kg of body weight per day or 0.02 mg/kg of dry matter [101]. The occurrence of this toxic compound in heat-treated foods is high. It is found primarily in edible oils and fats or products made from them, such as toast, crackers, cereals, margarine, milk, potato chips, doughnuts, and infant products such as infant formula. 3-MCPD is known to pass through breast milk, as it has been observed in the milk of lactating women who have eaten foods containing this compound. The main toxic metabolites are β-chlorolactaldehyde and β-chlorolactic acid, which can cause testicular and renal toxicity when ingested. The β-chlorolactaldehyde inhibits enzymes involved in glycolysis. On the other hand, the β-chlorolactic has oxalic acid as a metabolite that influences the renal system [102].

The powdered infant formula industry often uses vegetable oils such as palm oil or palm olein, which are subjected to heat treatments during their manufacture and storage. Recent analyses of infant formulas have reported the presence of 3-MCPD in various concentrations (Table 3), and mitigation measures have been initiated to reduce these levels. Analysis of infant food products from 3 different manufacturers sold in Prague [103] found esterified 3-MCPD. The authors note that babies have a high energy requirement but are limited in the volume of food that can be digested. They maintain that the only source of large amounts of energy in a formula is fat, so there is a direct relationship between the content of esterified 3-MCPD and the fat content of the formula, consistent with the high levels of 3-MCPD found in infant formulas.

In the United States, mitigation efforts between 2013 and 2019 had some impact on the amount of 3-MCPD in infant formula produced by some manufacturers, but overall levels of this undesirable toxic compound remained high for other manufacturers [104,105,106,107,108].

**Table 3:** Studies that report content of 3-Monochloro-1,2-Propanoldiol (3-MCPD) in infant formulas.

Research Objective	Infant Formulas and Method of Analysis	Findings	Country of Study
Investigate the concentration of bound 3-MCPD in infant formula available in retail markets in Prague.	14 infant and baby food products from 3 manufactures in outlets in Prague. GC-MS.	Bound 3-MCPD content ranged from 1042 to 2060 g/kg. There is a direct relationship between the content of 3-MCPD and the fat content. Vegetable oils used by manufacturers often contain elevated levels of 3-MCPD depending on their origin and other factors.	Czech Republic, 2008 [103]
Determination of 3-MCPD and 2-MCPD esters in infant formulas	88 infant formula samples GC-MS	The mean concentration range was 41 µg/kg for 2-MCPD and 185 µg/kg for 3-MCPD. This indicates a possible health risk for infants.	China, 2016 [109]
Analyze the occurrence of 3-MCPD and glycidiol esters in infant formulas in US stores.	98 powdered and liquid infant formulas purchased between 2013 and 2016. GC-MS	Results ranged from 0.072 to 0.16 mg/kg for 3-MCPD and 0.005 to 0.15 mg/kg for glycidiol. Formulas that did not contain palm oil/olein had lower concentrations.	USA, 2017 [104]
Validate a GC and mass spectrometry method as a method of simultaneous analysis of 3-MCPD and glycidiol esters in powdered infant formulas from Brazilian stores. Evaluate the potential risk associated with these contaminants in infant formulas.	Starter and follow-up formulas with cow’s milk and soy: with prebiotics, nucleotides and essential fatty acids from 4 different manufacturers in São Paulo Brazil in 2015. GC-MS	Results ranged from non-detectable to 0.60 mg/kg for 3-MCPD and non-detectable to 0.75 mg/kg for glycidiol esters. Preliminary exposure assessment showed intake for 3-MCPD to be 5.81 mg/kg and for glycidol esters to be 10.46 mg/kg per day, considering the 95th percentile. A potential health risk to consumers of these products was raised and should be monitored.	Brazil, 2017 [75]
Assess infant formulas from the previous (2013–2016) study to see if manufacturers had implemented mitigation strategies to improve contaminant concentration levels.	222 infant formulas purchased (2017 to 2019) from 4 different manufacturers. GC-MS.	3-MCPD levels ranged from 0.035 µg/g to 0.63 µg/g, and bound glycidol from 0.019 µg/g to 0.22 µg/g. There were improvements in concentration levels due to mitigation methods in palm oil-based formulations. However, some products that did not apply the mitigation strategies continue to present high levels of 3-MCPD.	USA, 2020 [106]
The data produced in this study were assessed to determine if 3-MCPD and/or glycidyl ester contents varied in formulas produced by different manufacturers. In addition, data for the 2019 formulas were compared to occurrence data for German formulas purchased in 2015 to determine whether manufacturers have improved mitigation strategies for minimizing MCPD and glycidyl ester levels over the 4-year period.	45 infant formula products produced by 8 different manufacturers and purchased from German supermarkets. GC-MS	43 infant formulas contained palm oil, and their 3-MCPD and glycidyl ester contents were consistent with the individual esters typically found in this type of oil. 3-MCPD levels were found to be similar for the German formulas (0.054 mg/kg) and the U.S. formulas (0.077 mg/kg) purchased during the same period. The contaminant levels across all formulas analyzed were exceptionally low (in comparison to bound 3-MCPD and glycidol concentrations in refined oils reported in previous studies).	Germany, 2021 [107]
Evaluation of 3-MCPD ester consumption levels in breast milk and infant formula	30 samples of breast milk and 23 samples of infant formulas GC-MS/MS	The estimated average daily intake of 3-MCPDE from breast milk in infants aged 0 to <12 months ranged from 2.38–3.48 μg/kg bw/day. The mean values for 3-MCPDE intake from infant formula were 2.34, 0.26, and 0.24 μg/kg bw/day at 0–6, 6–12, and 12–24 months, respectively.	China, 2022 [110]
Risk assessment for infants from 3-MCPD in infant formulas	108 formula samples GC/MS	The range of 3-MCPD concentration in infant formulas was 4.0–13.0 µg/kg. Rigorous control is necessary in the manufacture of infant formulas.	Iran, 2025 [111]

(GC-MS. Gas Chromatography Mass Spectrometry).

In Brazil, infant formulas using cow’s milk and soy as raw materials for their manufacture and enriched with prebiotics, nucleotides, and essential fatty acids were analyzed. Results for 3-MCPD content ranged from undetectable to 600 µg/kg. The exposure assessment showed that a child’s intake could be 5.81 mg/kg per day, posing a potential risk to their health [75]. Given the health problems expected from a daily intake of 3-MCPD in children who consume these infant formulas, various mitigation strategies are needed to reduce their exposure to this compound.

Intestinal permeability is an important factor in the movement of substances across the enterocyte membrane and its subsequent effect on the organism. Araujo et al. (2020) evaluated the intestinal permeability of 3-MCPD and its monoester derivatives present in infant formulas, using an in vitro model of polarized Caco-2 cells [112]. Their results showed that the monoesters were hydrolyzed to 3-MCPD and that the initial 3-MCPD easily enters through the Caco-2 cell monolayer. Abraham et al. (2021) quantified 3-MCPD in urine before and after consumption of different edible oils in a human study [113]. Their results indicated that, after intestinal absorption, the elimination of 3-MCPD via the kidneys was 5.8 h and the half-life was 3.6 h, reflecting a rapid metabolism.

**Figure 7:**
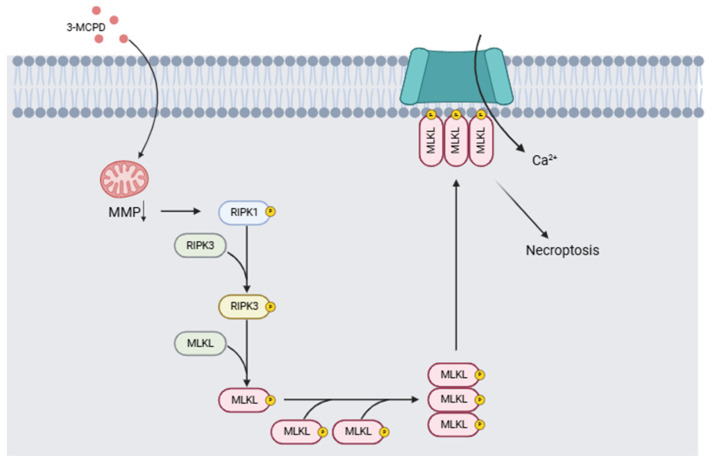
Mechanism of action induced by 3-MCPD. It causes a decrease in mitochondrial membrane potential (MMP), which triggers the activation of the RIPK1/RIPK3/MLKL signaling pathway involved in necroptosis processes (Modified from [115,119]). (Image created with BioRender.com).

The health problems linked to exposure to 3-MCPD include its nephrotoxicity and its carcinogenic and/or genotoxic potential. In two different studies, Huang et al. [114] compared the effects of a single dose of 3-MCPD dipalmitate vs. the same volume of olive oil on mice of different strains. Twenty-four hours after administration, the mice were decapitated, and the tissues were collected for study. Necroptosis was demonstrated in acute kidney injury induced by 3-MCPD, and induction of proinflammatory cytokines by necroptosis was detected. The authors conclude that 3-MCPD can induce renal toxicity, inducing tubular cell death by activating the RIPK1/RIPK3/MLKL-related necroptosis pathway, generating acute kidney injury, and activating the programmed cell death pathway of tubular cells in kidney tissue. Liu et al. [115] proposed a mechanism of toxicity associated with 3-MCPD and glycidol exposure based on toxicity results in in vitro and in vivo models. Their results indicate that 3-MCPD induces the activation of necroptosis in the in vitro model following mitochondrial damage, leading to the activation of the RIPK1/RIPK3/MLKL signaling pathway (Figure 7). Acute renal failure and carcinogenicity secondary to the renal lesions have also been reported [116]. Barocelli [117] administered equimolar doses of 3-MCPD of 29.5, 7.37, and 1.84 mg/kg per day over 90 days to male and female rats (n = 10 per group) while monitoring exposure and bioavailability. Histopathological examination confirmed that the kidney was tubulotoxic, contributing to acute renal failure in 20 to 50% of animals given doses of 29.5 mg/kg per day. Males exposed to these doses developed extensive testicular toxicity. Kidney and testicular damage occurred at lower doses from 5.6 and 8.4 mg/kg, thus confirming that the main target organs are the kidneys and testicles. Mossoba et al. [118] studied renal HK-2 cells of the proximal tubule in vitro. However, the authors posit that adult proximal tubule cells are not as vulnerable as rodent cells used in previous experiments and point out a mismatch in vitro and in vivo conditions, where 3-MCPD toxicity depends on their metabolism in vivo. These studies highlight the inherent vulnerability of the organs of developing infants and young children.

### 3.3. Attempted Adulteration and/or Fraud

#### Melamine (MEL) and Cyanuric Acid (CYA); CID:46878591

Melamine is a highly polar basic compound (Figure 2d). Cyanuric acid is a structural analog of melamine and is a weak acid generated as a metabolite of melamine [120]. The neutral forms of melamine and cyanuric acid form high molecular weight complexes linked by hydrogen bonds called melamine cyanurate, forming aromatic rings that stack up to form crystals [121]. Upon ingestion, most melamine is excreted unmetabolized by mammals. Because of the nitrogen atoms contained in its chemical structure, melamine can project the protein content of a substance higher than its real value. Thus, it may function as an adulterant, and its presence has been reported in various foods, such as pet food and infant formulas. However, it has been suggested that intestinal bacteria may metabolize some of the melamine to cyanuric acid, with a negative health impact [122]. The formation of melamine and cyanuric acid crystals in the kidneys significantly increases their toxicity. However, when these compounds are found separately, they are not considered highly toxic, with a lethal dose 50 (LD50) of 3.1 g/kg and 7.7 g/kg, respectively, values measured in rats and with a melamine half-life of 2.7 h [123]. Zhu & Kannan (2019) determined the basal levels of melamine and cyanuric acid in human urine [124]. Their results indicate that the average concentration of cyanuric acid was 16 ng/mL while that of melamine was 3.3 ng/mL. The deaths of six Chinese children from nephrolithiasis have been linked to infant formulas adulterated with melamine, a compound that, when combined with cyanuric acid, results in the formation of crystals that cause kidney stones, and irreversible kidney damage [33]. In addition to the observed nephrotoxic effect, toxic effects are described during the embryonic period in animal models exposed to the melamine-cyanuric acid complex. A decrease in fetal and placental weight and delayed fetal ossification have also been reported [125].

**Figure 8:**
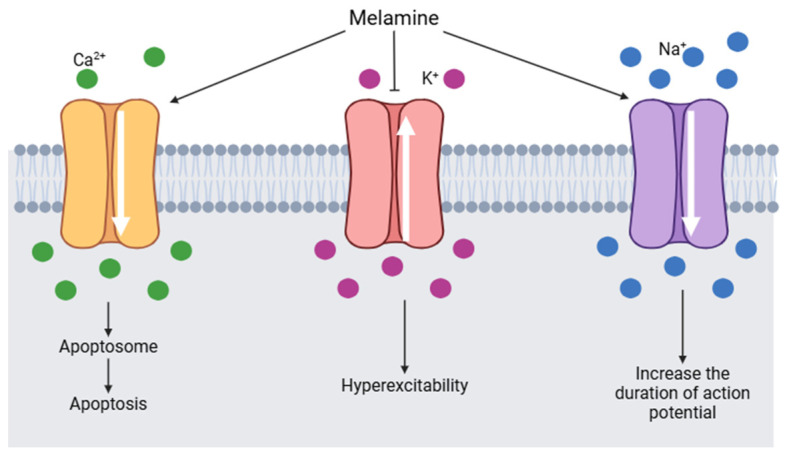
Changes in voltage-gated channels induced by melamine (modified from [126]) (Image created using BioRender.com).

It is estimated that melamine could interact with voltage-gated sodium channels (VGSCs). VGSCs are responsible for initiating and propagating the action potential in hippocampal neurons. By binding to a segment of the β subunit of VGSCs, melamine causes an alteration in the flow of calcium, potassium, and sodium ions (Figure 8). Sodium channels are activated and the duration of the action potential increases. Calcium channels are also activated by melamine, consequently, the intracellular calcium concentration increases and induces the formation of apotosomes. At the same time, potassium channels are blocked by melamine and generate hyperexcitability [126].

At the renal level, melamine is involved in the activation of cyclooxygenase-2 (COX-2) and PGF2α. It also regulates the activation of fibronectin, TGF-β1, and BMP4 in the kidney [127]. In addition, it has been observed that inflammation and increased melamine induce the activation of the NF-kB signaling cascade, positively regulating the expression of NOX, an enzyme involved in the increase in ROS production [128]. These processes contribute to renal alterations due to prolonged exposure to melamine.

In several countries, the melamine content of infant formulas was analyzed to standardize quality criteria and improve detection methods for this undesirable compound (Table 4). Chinese investigators collected samples of infant formula from markets in Gansu and Hebei provinces in Beijing, including those that the families of affected children had purchased. For comparative purposes, 38 samples from outside the Sanlu Group, China’s state-owned dairy company, were also analyzed, along with 87 raw material adulterants. Of the infant formulas analyzed, 87 tested positive for melamine, 51 with a concentration greater than 1000 mg/kg. The lowest concentration was 118, and the maximum was 4700 mg/kg. Thirty-one samples with high concentrations were found within the raw materials, coinciding with those from the Sanlu Group. The presence of melamine as an adulterant in infant formulas was confirmed and a provisional control limit of 1 mg/kg in infant formula and 2.5 mg/kg in other foods was established in China [33]. These limits were adopted in Europe, the United States, and Canada. Subsequently, a similar health alert was activated in other parts of the world, such as Switzerland and Canada, in response to the melamine and cyanuric acid found in some products at doses that exceeded the toxic limits established by the WHO [129,130]. Tittlemier et al. in 2009 analyzed 94 infant formula products purchased at major retail stores in Ottawa for immediate availability: 31 liquid and 63 powdered formulas, including soy-based milks. Melamine was detected in 71 of the 94 infant formulas in concentrations ranging between 4.31 and 346 mg/kg [130]. In a second study in Canada, a different methodology (UPLC-MS/MS) was used to analyze 94 milk-based, soy-based, iron-fortified, and calcium-fortified formulas. The presence of melamine and cyanuric acid was reported in all the formulas analyzed. However, average concentrations were generally low <0.5 mg/kg [131]. In China, Meng et al. (2015) developed a method for rapid screening and quantification of cyromazine and its metabolites melamine, ammelide, ammeline, cyanuric acid, and dicyandiamide (DCD) residues in infant milk powder samples by Ultra-performance Liquid Chromatography (UPLC) coupled with Quadrupole Time-of-flight Mass Spectrometry (QTOF-MS) and Triple Quadrupole Mass Spectrometry (MS/MS) with electrospray ionization (ESI) mode, using 8 samples of powdered milk for infants from 6–12 months and from 1–3 years of age [132]. The content of cyromazine in all 8 samples and melamine in 4 samples were in the range of 3.5–45 μg/kg and 8–25 μg/kg, respectively. Apparently, although authorities have imposed limits on these compounds, their presence in infant formulas continues. The consumption of melamine in food can cause damage to the central nervous system and kidneys. In the brain, it causes a loss of volume of the hippocampus, initiated by the activation of oxidative stress, and, as a consequence, triggers a deterioration in cognitive function. Insoluble crystals known as melamine cyanurate are formed in the kidneys, which promote the agglomeration and growth of kidney stones [126]. In addition, it alters the kidney’s ability to concentrate urine and promotes the development of obstructive nephropathy [127]. It is important to establish the effect of melanin and cyanuric acid on children’s health to prevent possible damage to the urinary tract and central nervous system, for example. Sathyanarayana et al. (2019) evaluated the relationship of melanin and cyanuric acid to the development of early kidney injury in children aged 4 months to 2 years [133]. Their results showed a statistically significant increase in kidney injury molecule 1 (KIM1) related to exposure to cyanuric acid, but not related to melanin. Therefore, KIM1 could be used as an indicator of nephrotoxicity in early kidney injury. In another study, Sun et al. (2022) evaluated the cytotoxic effect of melanin and cyanuric acid in an in vitro model of hippocampal neurons and an in vivo model to assess ROS production and its effects [134]. Their results indicate that the combination of 10 µg/mL melanin and 10 µg/mL cyanuric acid induces the apoptosis process mediated by the increase in ROS levels and the activation of caspase-3, causing a deterioration in neuronal activity and synaptic plasticity.

## 4. Discussion

The early stages of human development represent a sensitive and vulnerable window in which the organism reacts and changes according to stimuli that are presented to it. UNICEF [137] indicates that the nutritional status of 200 million children worldwide is compromised by the quality and quantity of their food. One of these challenges occurs when children are fed formula instead of breast milk [138,139]. There are two aspects to this challenge. The first is that in their present state, such formulas cannot provide the nutritional equivalent of breast milk, a dynamic fluid that changes its composition throughout the feeding and meets the nutritional requirements of the newborn during their first six months. The second is that these formulas may contain a variety of undesirable compounds, not only posing a possible risk during infancy but also a potential increase in the infant’s susceptibility to diseases or other problems later in life. The possible risks to the health of infants who consume infant formulas are related to predisposition to the liver, kidney, cardiovascular, neurological, motor, developmental, reproductive, cancer development damage, and food allergies.

It is the responsibility of food science and technology to be aware of these challenges and work towards reducing the risks currently posed to infants, whether by the nutritional shortcomings of current infant formulas or the presence of a variety of undesirable compounds present in the formulas or resulting unintentionally from chemical processes present during their formulation. Most of the toxicological studies reviewed above involve laboratory animals, in vitro studies, and reports in adults, except those on melamine, which reflect the effects of consumption by infants. In the case of animal studies, these have been carried out with pure compounds and at certain concentrations that may not have the same effect as when present in infant formulas at much lower concentrations.

With these previous studies, we can estimate the possible potential effects of consuming HMF, acrylamide, 3-MCPD, melamine, and cyanuric acid present in infant formulas, including the development of adenomas, deterioration in liver functions, activation of oncogenes, neurotoxicity, and kidney damage. Martins et al. [140] mention other related symptoms, including irritation of the mucous membranes, skin, respiratory system, and eyes, as well as the possible development of neurodegenerative diseases, cardiovascular diseases, and diabetes, effects that could manifest in the long term. However, there are no studies that establish a relationship between the dose consumed in infant formulas and the appearance of short-term diseases in young children, which makes it difficult to evaluate the long-term impact. Therefore, it is essential that monitoring strategies continue to be developed to protect children’s health and reduce their exposure. However, it is also important to note that exposure of newborns to these compounds is not ideal and may represent a potential health risk to infants whose immunological immaturity and inability to detoxify makes them uniquely vulnerable to their potentially harmful effects. Therefore, HMF, acrylamide, 3-MCPD, melamine, and cyanuric acid in infant formula should be monitored through long-term epidemiological studies and new toxicological studies focused on infants should be conducted, in order to more thoroughly assess the risks associated with their presence in the body, providing guidelines to ensure food safety and protect infant health. The food industry must conduct further research into the production of these compounds in the manufacture of infant formulas, and impose much stricter regulatory measures throughout the powdered milk production chain to ensure the safety of infant formulas. Likewise, faster and more sensitive methods must be developed for detecting undesirable compounds and incorporating them as routine testing methods in the infant formula production chain. Finally, governments must allocate more resources and improve current legislation to promote infant feeding policies and the continuous monitoring of infant formulas. All of this underscores how problematic the direct extrapolation of the effects of chronic consumption of these components at low levels is, and complicates efforts to establish well-defined permissible limits in infant formulas. Nevertheless, this uncertainty is further reason to allow only the lowest possible level of such compounds in infant formulas.

Recent literature is encouraging in this respect. For example, mitigation strategies have been undertaken to significantly reduce levels of advanced glycation products, including Maillard reaction products, including ultraviolet irradiation, the addition of phenolic compounds, resveratrol, and other phytochemicals [141,142,143], and the use of non-thermal treatments such as high hydrostatic pressure processing as an alternative to pasteurization [144], or removal methods [145]. At the same time, detection methods for melamine and cyanuric acid have improved in terms of efficiency and speed. As a preventive measure, these may reduce exposure to HMF, Acrylamide, 3-MCPD, Melamine, and cyanuric acid compounds that have been reported in infant formulas and some infant products [146]

Concerning the products of vegetable oil refinement, 3-MCPD, mitigation methods involving chlorine donor compounds for the formation of MCPD esters in food and petroleum products have been tested. Inorganic and organic chlorinating agents such as NaCl and Lindane have been evaluated as a practical method of mitigation in oil and food products. Absorbent materials such as calcined zeolite and magnesium silicate are also reported, which may remove up to 40 % of glycidiol esters from refined oils, and food products in general [147]. However, trace-level residues from these mitigation processes must be addressed. In summary, the primary goal of such mitigation measures should be to minimize exposure to toxic compounds in formula-fed infants to guarantee a better state of health during infancy and minimize the predisposition to morbidities or diseases in later stages of life.

## Figures and Tables

**Table 1:** Studies reporting HMF content in infant formulas.

Research Objective	Infant Formulas and Method of Analysis	Findings	Country of Study
To determine the content of furfural compounds in initial and follow-up infant formulas stored at 20 and 37 °C for two years.	Initial and follow-up infant formulas RP-HPLC	Free HMF levels were related to storage time. Higher content of HMF in the powder samples than in the liquid ones. Positive correlation between HMF and storage temperature (20 and 37 °C). No significant variations under refrigeration.	Spain, 2002 [55]
Continue evaluating the furfural compounds in the initial and follow-up formulas of the previous study during the second year of storage. Relate the furfural content obtained with the available lysine values.	Initial and follow-up infant formulas RP-HPLC	The initial and follow-up infant formulas showed a similar behavior during shelf life, with a significant increase in furfural content at the end of the storage period which was more marked at 37 °C than at 20 °C. HMF + F (2-furylmethylketone) during the second year was higher in the follow-up infant formula. In both infant formulas, a correlation was obtained between the furfural contents and the cubic time variable. This explains the irregular increase in HMF over time, and between available lysine and furfural compounds in the second year of storage. This indicates that the Maillard reaction is in an advanced state.	Spain, 2005 [54]
Develop a RP-HPLC-DAD method to evaluate furfural compounds in milk-based formulas. Obtain more information on the formation of furfurals, the stability of milk-based formulas, and the usefulness of analysis of furfural compounds to assess spoilage in these products.	Experimental milk-based formula powder stored at 25 and 37 °C from production until 15 months RP-HPLC-DAD	The RP-HPLC-DAD method was relatively simple and reproducible for measuring furfural compounds in milk-based formulas. HMF was the main furfural compound detected in the formula followed by F. Furfural levels were higher in formula stored at 37 °C than at 25 °C. The evaluation of HMF is an indicator of the Maillard reaction in this type of product and can help to assess the stability of the product throughout its shelf life.	Spain, 2005 [32]
Determining HMF and F contained in follow-on milk and infant formulas consumed in Ankara, Turkey.	60 follow-on milks and 49 powdered infant formulas. Milk-based and cereal-based formulas exposed to 25 °C RP-HPLC	Potential HMF and F (minimum and maximum) 247.00 to 2924.5 and 5.87 to 40.99 µg/ 100 g. All samples had presence of HMF. Higher content of HMF and F was found in the dates close to expiration.	Turkey, 2015 [56]
Evaluate the content and evolution of potential furfurals (HMF, F, FMC and MF) in commercial infant formulas for 70 days after package opening.	20 powdered infant formulas from France, Mexico, Denmark, Spain and the UK. RP-HPLC-DAD	The increase in furfurals was analyzed within the first 3 to 5 months of shelf life of infant formulas from opening the package up to 70 days after. Average values of potential HMF: at the beginning 1115.2, at 30 days 1157.6 and at 70 days 1344.5 µg/ 100 g showing that the Maillard Reaction increases after opening the packages. Potential HMF intake by a 6-month-old formula-fed infant was estimated to be between 630 µg/ 100 g and 3250 µg/ 100 g per day.	Spain, 2015 [11]
Find relationships between the ingredients and thermal treatment used in the production of infant formulas in order to evaluate the nutritional value and the possible toxic effect of different types of infant formulas on the Spanish market.	Thirteen commercial powdered infant formulas: eight adapted and five follow-ups (hypoallergenic and soy-based) in various local markets. HPLC	HMF values ranging from not detected to 11.7 mg/100 g protein. Low thermal damage was observed specifically speaking of HMF. However, the expression of furosine was at a high level.	Spain, 2017 [57]
Evaluate the quality of infant formulas supplemented with prebiotics and without prebiotics through thermal indices, furosine and determination of free HMF, as well as the influence of storage (8 to 15 months) by the Maillard Reaction.	Start and follow-up formulas ion-pair RP-HPLC	Furosine and HMF content: 94 to 1226 and 315 to 965 mg/100 g, respectively. No significant statistical differences between formulas supplemented and not supplemented with prebiotics. Storage for 15 months produced an increase in furosine. The increase in HMF was in the range of 62-510 µg/100 g. This was attributed to excessive heat treatment during processing, storage, or ingredients used in its manufacture.	Spain, 2018 [58]
Investigate physicochemical changes in infant formulas at different storage temperatures (10, 20, 30 and 40 °C) for 6 months.	Ready-to-drink liquid infant formula stored for 6 months. Using the slightly modified Kenney and Baset method.	Changes in the physicochemical properties during storage of 6 months at different temperatures. HMF increased with storage time. Greater significant increase at 40 °C, more redness and browning in the presence of HMF. Physicochemical changes were accelerated by the Maillard Reaction at higher storage temperatures.	Korean, 2018 [59]
Evaluate the content of minerals, toxic metals and HMF in foods and infant formulas. Shed light on the potential effects of undesirable substances on infants as a vulnerable group.	6 starter and follow-on infant formulas Spectrometry	HMF in infant formula ranged from 0.29 mg/kg to 7.87 mg/kg, significantly increasing after storage at 30 °C for 21 days from 1.80 mg/kg to 9.43 mg/kg.	Malta, 2019 [60]
Evaluate the content and factors such as fat percentage, the presence of lactose or products of its hydrolysis and type of preservation process affecting the concentration of 5-HMF in cow’s milk and modified milk for infants.	Product intended for newborns and infants up to 6 months of age in Chromatographic analysis was performed on a Merck Hitachi HPLC system	The average content of 5-HMF in infant milk powder was 2.3 mg kg, that is about 314 μg L^−1^	Poland, 2020 [61]

(RP-HPLC reversed-phase high-performance liquid chromatography; RP-HPLC-DAD High-performance liquid chromatography with diode array detector (DAD) is the most useful method for identification and quantification of antioxidant vitamins in biological fluids; HS-SPME-GC-MS Headspace solid-phase microextraction coupled to gas chromatographymass spectrometry).

**Table 2:** Studies reporting acrylamide in infant formulas.

Research Objective	Infant Formulas and Method of Analysis	Findings	Country of Study
Improve acrylamide intake limits in children as opposed to adults. Establish the development and validation of an improved analytical method.	Infant formulas LC-MS-MS and GC-MS.	Acrylamide was found in 5 out of 8 infant formulas.	Sweden, 2005 [79]
Determine acrylamide levels in baby food products. To assess dietary exposure to acrylamide in non-breastfed infants 6 to 12 months of age.	12 follow-up infant formulas LC-MS/MS	The minimum exposure range for infants was 0.41 to 0.62 μg/kg/day, while the maximum exposure level was 7.47 to 12.35 μg/kg body weight/day. This is a high exposure to acrylamide, considering that it is such an early stage of life.	Polan, 2012 [80]
To determine the acrylamide content in Colombian foods (including infant formula)	9 infant formulas GC/MS	In 5 samples of infant formula, levels were below LOD; in the other 4 samples, the LOQ for acrylamide was 1821 µg/kg.	Colombia, 2015 [83]
To quantify the concentration of acrylamide in foods consumed by infants and young children	14 follow-up formula and 10 infant formula LC-MS/MS	Range of 0.14–2.2 µg/kg and 0.60–2.9 µg/kg of acrylamide, in follow-on formulas and infant formulas, respectively. This is related to the processing conditions of the product.	France, 2018 [84]
To assess dietary exposure to acrylamide, furans, and polycyclic aromatic hydrocarbons, and their association with health risk in non-breastfed children under 3 years of age living in France.	Cross-sectional survey, collection of food samples for 3 consecutive days of consumption products in non-breastfed children from month 1 to 36. The most frequently consumed foods in various presentations were analyzed.	Mean daily acrylamide exposure ranged from 0.141 µg/kg in children aged 1 to 4 months, to 0.708 µg/kg in children aged 13–36 months. The 90th percentile ranged from 0.372–1.60 µg/kg. Reference value 0.2 µg/kg. In this study, acrylamide was not specifically reported in infant formulas, but after its preparation, its levels were evaluated to study an approximate infant exposure. The important of this study is that it shows that the main source of acrylamide for newborns is infant formula.	France, 2019 [81]
Twenty-seven samples of powdered infant formula, including three brands for three age groups, were analyzed for their acrylamide level.	ME-GC-MS analytical technique	The concentration ranges in products for babies 0–6 months were between 48 and 3191 µg/kg, while products for babies 6–12 months contained between 918 and 5835 µg/kg of acrylamide. Products developed for babies 12–24 months showed between 1290 and 4400 µg/kg. The mean total acrylamide concentration for products for infants 0–6, 6–12, and 12–24 months was 992, 2349, and 2372 µg/kg, respectively. In products for infants 0–6 months, the maximum gap was observed (between 992 and 2349 µg/kg acrylamide).	Iran, 2021 [82]
To determine the levels of acrylamide in different frequently consumed and highly popular commercial brands in different periods of time.	6 infant formulas LC-MS/MS	The mean acrylamide concentration was 45.1, 62.5 and 88.9 µg/kg at 0–5, 6–12 and >12 months, respectively. Findings were useful for evaluating infant formulas to reduce acrylamide consumption.	Turkey, 2022 [85]

(LC-MS/MS Liquid chromatography-mass spectrometry; GC-MS Gas chromatography-mass spectrometry).

**Table 4:** Studies reporting melamine and cyanuric acid content in infant formulas.

Research Objective	Infant Formulas and Method of Analysis	Findings	Country of Study
Evaluate the use of near-infrared techniques to quantify and detect melamine in powdered infant formula.	94 samples of infant formula from retail stores. LC-MS/MS	Melamine was found in 71 infant formulas in concentrations from 4.31 to 346 μg/kg with an average value of 16 μg/kg. WHO recommendation of 200 µg/kg/day.	Canada, 2009 [130]
Report the results of the analysis of Sanlu infant formulas during the 2008 melamine crisis.	111 samples of infant formula from the Sanlu Group (including families with affected children), in addition to 38 infant formulas from markets and 87 raw materials used for adulteration of infant formula. LC-MS/MS.	A high prevalence of elevated melamine concentrations was found in the Sanlu Group infant formulas, suggesting attempted adulteration. This was the cause of the epidemic of stones in the urinary tract in China.	China, 2009 [33]
Determine melamine, ammeline, ammelide and cyanuric acid in infant formula purchased in Canada, using liquid chromatography-tandem mass spectrometry	94 infant formulas purchased from retailers in Ottawa: liquid, powder, milk-based, soy-based, iron-fortified and calcium-fortified. UPLC-MS/MS.	Melamine and cyanuric acid were detected in almost all infant formulas. Concentrations were generally low <0.5 mg kg. The highest concentrations observed were 0.32 mg/kg melamine and 0.45 mg/kg cyanuric acid.	Canada, 2011 [131]
To determine the presence of melamine and cyanuric acid in different foods (powdered infant formula)	LC-MS/MS	A melamine concentration of 8.7 mg/kg was found for powdered milk.	USA, 2011 [135]
Develop a method for rapid screening and quantification of cyromazine and its metabolites melamine, ammelide, ammeline, cyanuric acid, and dicyandiamide residues in infant milk powder samples by UPLC-QTOF-MS and UHPLC-ESI-MS/MS	8 samples of powdered milk for infants from 6 to 12 months and from 1 to 3 years of age. UPLC-QTOF-MS UHPLC-ESI-MS/MS	The content for cyromazine in all 8 samples and melamine in 4 samples were in the range of 3.5–45 μg/kg and 8–25 μg/kg, respectively.	China, 2015 [132]
To determine the concentration of melamine and cyanuric acid in powdered dairy products (including infant formulas)	30 samples of powdered infant formula LC-MS/MS	The limit of detection (LOD) for melamine and cyanuric acid is 0.25 mg/kg. Melamine was found to be present in 67.7 % of the samples, while cyanuric acid was not detectable. Melamine and cyanuric acid should be monitored in infant formulas.	Egypt, 2022 [136]

(LC-MS/MS Liquid chromatography-mass spectrometry; UPLC-MS/MS Ultra performance liquid chromatography-tandem mass spectrometer; UPLC-QTOF-MS Ultra-high performance liquid chromatography with quadrupole time-of-flight mass spectrometry; UHPLC-ESI-MS/MS rapid ultra-high performance liquid chromatography-electrospray ionization tandem mass spectrometry).

## Data Availability

Not applicable.
